# Evaluation of “Colorful Life”: A Multi-Addiction Expressive Arts Intervention Program for Adolescents of Addicted Parents and Parents with Addiction

**DOI:** 10.1007/s11469-018-9899-3

**Published:** 2018-06-05

**Authors:** Hildie Leung, Daniel T. L. Shek, Lu Yu, Florence K. Y. Wu, Moon Y. M. Law, Elda M. L. Chan, Camilla K. M. Lo

**Affiliations:** 1 Department of Applied Social Sciences, The Hong Kong Polytechnic University, Hong Kong, People’s Republic of China; 2 Integrated Centre on Addiction Prevention & Treatment, Tung Wah Group of Hospitals, Hong Kong, People’s Republic of China

**Keywords:** Multi-addiction prevention, Expressive arts, Intervention, Addicted parents, Adolescents

## Abstract

This study evaluated an expressive arts intervention program (“Colorful Life”) for adolescents with addicted parents and parents with addiction in Hong Kong. Different evaluation strategies were employed. Objective outcome evaluation adopting a one group pretest-posttest design showed positive changes in adolescents’ (*N* = 43) beliefs about addiction. Both adolescents’ and parents’ (*N* = 21) psychosocial competencies were enhanced post-intervention. Subjective outcome evaluation from both adolescent (*N* = 47) and parent (*N* = 22) groups showed positive perceptions of the program content, implementers, and achievement of program objectives. Findings provided preliminary evidence to support and yielded practical implications for the adoption of the multi-addiction syndrome model, positive youth development, and expressive arts approaches in the development of interventions for high-risk adolescents and addictive parents.

In the new edition of Diagnostic and Statistical Manual, 5th edition (DSM-5; American Psychiatric Association 2013), a revised category “Substance-Related and Addictive Disorders” was proposed. This new category combines two general types of addiction disorders, i.e., substance-related addiction (e.g., drug and alcohol) and behavioral addiction (e.g., gambling, Internet, and sex). The adoption of this multi-addiction approach was supported by empirical evidence. Villella et al. (2011) identified strong correlations among various addictive behaviors including pathological gambling, Internet, work, and exercise addiction. These findings suggested the presence of a common psychopathological dimension underlying various addictive behaviors. Functional similarities between substance-related and behavioral addictions at the biological, psychological, and social levels have been observed (Grant et al. 2006; Holden 2001). Scholars have thus warned about the likelihood of individuals to present two or more addictions at the same time, often known as multiple addictions (Miller et al. 1990; Pallanti et al. 2006). As such, integrated multi-addiction treatment models have gained their popularity in the West. However, few multi-addiction prevention or intervention programs have to date been developed and implemented in non-Western contexts.

Earlier studies in the field have predominantly examined the adverse impact of addiction on the addicts (e.g., Crawford et al. 2003). More recently, scholars have reported the potential detrimental effect of addiction on the addicts’ families and offsprings. Studies have demonstrated the negative influences of substance use disorders on parental self-efficacy (Raynor 2013), self-esteem (DiLorenzo et al. 2001), and parenting attitudes (Suchman et al. 2013). In Hong Kong, Chan et al. (2016) found that the majority of the family members of problem gamblers reported a medium level of psychological distress, fair or poor health, and neither good nor bad quality of life. The impact of addicted parents on other familial characteristics have also been identified, such as low family cohesion and adaptability (Finzi-Dottan et al. 2006) and low paternal involvement (Fong and Lam 2007).

According to the National Survey on Drug Use and Health (NSDUH), approximately 8.3 million children under 18 years of age lived with at least one parent who abused illicit drugs or alcohol during the past 1 year (Office of Applied Studies 2009). The NSDUH data further revealed that adolescents living with parents who suffer from mental illness (AMI) and/or substance use disorders (SUD) are more likely to have SUD (Ali et al. 2016). Children and adolescents with addicted parents are not only more vulnerable to addiction (Chassin et al. 1993; Sher et al. 2005), but also to a vast range of mental disorders and deviant behaviors such as externalizing symptoms (Barnow et al. 2002), depression (Hill et al. 2008), and anxiety (Ebrahimi et al. 2015). Given the detrimental effects that the addicted parents may have on not only themselves, but also on their family and offspring, addiction prevention programs should target both the addicts and their family members, particularly their children. However, a review of the literature showed that these programs were developed and implemented in the West. Few of such programs were implemented in Hong Kong even though the need is apparent.

Recently, scholars and practitioners in Hong Kong have started to recognize the needs to develop intervention programs for adolescents with addicted parents based on the positive youth development approach with a focus on helping these youths flourish in spite of the adverse family circumstances. Chan et al. (2016) recommended that “skills enhancement programs and treatment groups in family functioning and family coping be developed… Treatment components should involve adaptation of healthy coping strategies, including self-care, interests building and expansion of social support” (p. 11). Generally, the studies in this area have pointed to two directions: (1) to enhance the adolescents’ personal strengths (Pearson et al. 2011; Ronel et al. 2011) and (2) to improve their problem-solving and help-seeking skills (Hall 2008; Kimiaee and Karimi 2015).

On the other hand, empirical evidence has suggested that the adverse effects of parents’ addiction on their child’s development can be moderated by positive parenting (e.g., consistency, appropriate discipline, and parental monitoring with parental warmth) (Molina et al. 2010). Researchers also found that addicted parents recognized that their drug abuse places their children at risk, and expressed a deep concern about their own children’s development (Haight et al. 2009). However, they reported struggles with effective discipline for their children (Slesnick et al. 2014) and shared that they wanted to learn how to discipline and manage their children’s misbehaviors, develop a closer relationship with their children, help their children to cope better with the family circumstances, and guide their children toward not using drugs or alcohol (Kerwin et al. 2014). Therefore, effective parenting skills are needed in an effective intervention program for parent addicts.

For patients with multi-addiction problems, art therapy is often part of a more varied program (Dickson 2007). Often, the patients perceived that art therapy provided a means to express and explore their emotions (Gallagher and Steele 2002; Horay 2006), interact with the group (Dickson 2007), and learn new skills to maintain sobriety (Horay 2006). Lev-Wiesel and Liraz’s (2007) study showed that drawings helped children (aged 9–14) of drug-addicted fathers better narrate their life experiences and be more able to reveal their emotions than the comparison group. This therapy provides opportunities to process grief and helps the youths take ownership of the therapeutic process. Moreover, because of the “cool factor” of the art therapy, it can also motivate the youth to participate in the therapy regularly, which then also affects the treatment outcome positively.

Against the above background, a multi-addiction intervention program entitled “Colorful Life” has been developed for both addicted parents and their adolescent children in Hong Kong based on expressive art therapy and principles of the positive youth development approach. Adolescent participants are guided through various expressive activities to discover their strengths and potential, develop self-regulatory skills, become more aware of their emotions, and learn to express their emotion in healthy ways. Other sessions focus on providing adolescents with possible help-seeking avenues, encouraging them to seek help when needed, and overcoming barriers in help-seeking. The participants are also taught to analyze different situations and equipped with problem-solving skills to find plausible solutions. For parent participants, the intervention program includes components to facilitate the parents to better understand the needs of children and adolescents at different developmental phases, gain positive parenting skills, and recognize the relationship between positive parenting (e.g., discipline, communication) and addiction. Via the use of art therapy, the addicted parents are encouraged to freely express their emotions and share their experiences with their counterparts and helping professionals so as to gain and provide support to one another. To evaluate the effectiveness of the program, both qualitative and quantitative studies have been conducted (Shek et al. 2017).

The present paper reports pioneer evaluation findings of this program.

## Method

### Participants and Procedures

Participants of the programs were recruited from January to July 2016. The social workers from the Integrated Centre on Addiction Prevention and Treatment sent out invitation letters to school social workers to invite students with addicted parents to participate. Parent participants (i.e., those suffering from addiction) were recruited via various organizations such as Cross Centre and Even Centre. A total of 47 adolescents and 22 parents participated in the program. While 43 adolescents (*M*_age_ = 16.28, SD = 1.80, Males = 29) and 21 parents (*M*_age_ = 48.76, SD = 10.12, Males = 13) participated in objective outcome evaluation, all participants responded to subjective outcome evaluation. The background information of the intervention programs is summarized in Table 1.

**Table 1:** Descriptive statistics of adolescent participants (*N* = 47) and parent participants (*N* = 22) of the program

	Adolescents	Parents
Mean age (SD)	16.28 (1.80)	48.76 (10.12)
Age range	13–20	32–64
Gender ratio (male/female)	29 (67.4%):14 (32.6%)	13 (68.4%):6 (31.6%)
Number (%) of participants		
Duration of the program	Adolescents	Parents
1 day		7 (33.3%)
5 days		5 (23.8%)
7 days		3 (14.3%)
8 days	13 (30.2%)	
10 days	4 (9.3%)	
15 days	5 (11.6%)	6 (28.6%)
21 days	6 (14.0%)	
50 days	8 (18.6%)	
78 days	7 (16.3%)	

Informed consent was obtained from the parents of the adolescents under the age of 16. For the parent participants, informed consent was obtained prior to the commencement of all evaluation procedures. Owing to the sensitive nature of the research topic, anonymity and confidentiality of the data collected were highly emphasized. The questionnaire was administered by trained and experienced research staff with sufficient time being provided for the respondents to complete the questionnaire.

### Evaluation Methods

To examine whether adolescent participants changed after participating in the program, objective outcome evaluation was conducted using a one-group pretest-posttest design. Participants completed the objective outcome evaluation form before the commencement of the first session and after the completion of the final session. In addition, to better understand students’ perceptions of the program, a subjective outcome evaluation form was administered to both parent and adolescent participants in the last session. Qualitative evaluation via focus group interviews was also conducted and the related findings will be published elsewhere. This research design has been extensively used for measuring the effectiveness of positive youth development programs as well as addiction prevention and intervention programs in Hong Kong (Shek and Sun 2013; Shek et al. 2016).

### Instruments

#### Objective Outcome Evaluation

Two objective outcome evaluation forms (for adolescent and parent, respectively) were developed based on the existing objective outcome assessments (e.g., Shek and Lam 2008; Shek et al. 2016; Young 1999) widely used in the positive youth development and multi-addiction prevention programs, literature review, and intended outcomes of the respective sessions of the present program. Experienced researchers and practitioners in the relevant fields were invited to review the items of the questionnaires and to comment on its relevance to the learning outcomes and clarity of the items. Minor revisions of wordings were made upon the feedback from the expert panel.

#### Adolescent Version

The adolescent version of the objective outcome evaluation was composed of 53 items tapping on five domains related to addiction as follows:

- *Domain A: Addictive Behavior* (8 items, pretest: *α =* 0.68 and posttest: *α =* 0.84*)*

The respondents indicated on a seven-point Likert scale the frequency that they engaged in the stated addictive behavior (e.g., smoking, consuming alcohol, substance use, gambling, and Internet use) in the past month. Options included 0 = *never*, 1 = *once*, 2 = *twice*, 3 = *three times*, 4 = *once per week*, 5 = *several times per week*, 6 = *daily*).

- *Domain B: Addictive Behavioral Intention* (8 items, pretest: *α =* 0.72 and posttest: *α =* 0.89)

To assess the adolescents’ addictive behavioral intention, the respondents were asked to indicate the likelihood that they would engage in the stated addictive behaviors in the coming 2 years. Options were anchored on a four-point scale ranging from 1 = *unlikely* to 4 = *definitely*. Items included “spending a considerable amount of time on the Internet or playing video games” and “participating in gambling activities”.

- Domain C: Psychosocial Competencies (10 items, pretest: *α* = 0.90 and posttest: *α* = 0.95)

The respondents’ psychosocial competencies were measured by asking them to specify their agreement on the items such as “When confronted with familial problems, I am not able to control my emotions”, “I am able to make use of my personal and social resources to strengthen myself”, and “I can overcome barriers to help-seeking”. The six-point Likert scale ranged from 1 = *strongly disagree* to 6 = *strongly agree*.

- Domain D: Knowledge on Addiction (10 items, pretest: *α =* 0.83 and posttest: *α* = 0.96)

The participants’ knowledge on addiction were tested using the three-choice items (*true*, *false*, *not sure*). Sample items included “Addictive behaviors may impact every family member” and “I am aware of different avenues to seek help”.

- Domain E: Beliefs about Addiction (17 items, pretest: *α* = 0.57 and posttest: *α* = 0.91)

The participants indicated their agreement on a scale ranging from 1 = *strongly disagree* to 6 = *strongly agree* to the statements regarding their beliefs about addiction such as “I understand that sometimes I cannot control my family member(s)’ addiction” and “Even though my parent is an addict, I can still have a positive identity”.

#### Parent Version

The parent version of the objective outcome evaluation was made up of 18 items tapping the parenting skills and practices (e.g., understanding the needs of their children, communicating with their children), as well as intrapersonal competencies (e.g., self-acceptance, the ability to self-reflect, problem-solving ability). The participants indicated their agreement to the statements such as “I understand the importance of maintaining a positive relationship with my children” and “I do not respect myself” (reversed scoring), ranging from 1 = *strongly disagree* to 6 = *strongly agree*. The Cronbach’s alpha for the pretest and posttest were 0.92 and 0.94, respectively.

#### Subjective Outcome Evaluation

The subjective outcome evaluation forms were revised based on a widely used program evaluation tool in Hong Kong and mainland China with high reliabilities (e.g., Shek et al. 2011, 2012, 2016). The outcome evaluation forms were divided into three main sections assessing the participants’ perceptions on (1) program attributes (10 items, adolescent *α* = 0.98 and parent *α* = 0.95); (2) implementer attributes (10 items, adolescent *α* = 0.97 and parent *α* = 0.96); and (3) program effectiveness (20 items for adolescent version, *α* = 0.99; 14 items for parent version, *α* = 0.96). The first two sections of the adolescent and parent subjective outcome evaluations were identical. Participants rated on a six-point Likert scale the extent they agreed on the items anchored at 1 = *strongly disagree* to 6 = *strongly agree*. The program effectiveness section was designed based on the intended program outcomes which are differed between the adolescent and parent versions. Participants were invited to rate on a five-point Likert scale the extent to which they perceived the program to have helped them achieve the stated outcomes. Options were anchored at 1 = *of no help* to 5 = *of great help*.

## Results

To gauge adolescent participants’ changes in terms of (1) addictive behaviors, (2) behavioral intentions, (3) psychosocial competencies, (4) knowledge about addiction, and (5) beliefs about addiction, paired sample *t* tests were conducted (see Table 2). Results revealed that the adolescents at posttest scored significantly higher on beliefs about addiction (*t* (42) = − 2.40, *p* < 0.05) while there were no significant differences in other indicators between pretest and posttest. However, a positive trend in the acquisition of psychosocial competencies was also observed.

**Table 2:** Mean scores (SD) of adolescent participants’ items tapping their main domains related to addiction at pretest and posttest periods, with results of paired sample *t* tests (*N* = 43)

	Pretest	Posttest	*t*
Addictive behaviors^a^			
Male	1.03 (0.59)	1.09 (1.15)	
Female	0.62 (0.87)	0.46 (0.89)	
Total	0.89 (0.90)	0.88 (1.10)	0.12
Addictive behaviors (categorical)^b^			
Male	0.26 (0.20)	0.30 (0.32)	
Female	0.19 (0.19)	0.12 (0.18)	
Total	0.24 (0.20)	0.24 (0.30)	− 0.19
Behavioral intentions^c^			
Male	1.58 (0.43)	1.71 (0.78)	
Female	1.29 (0.49)	1.33 (0.50)	
Total	1.49 (0.46)	1.59 (0.72)	− 1.21
Psychosocial competencies^d^			
Male	4.08 (0.65)	4.24 (1.03)	
Female	4.34 (1.15)	4.92 (0.81)	
Total	4.17 (0.84)	4.47 (1.00)	− 1.80*
Knowledge about addiction^e^			
Male	0.72 (0.25)	0.69 (0.41)	
Female	0.73 (0.33)	0.89 (0.22)	
Total	0.72 (0.46)	0.79 (0.69)	− 0.449
Beliefs about addiction^f^			
Male	3.85 (0.48)	4.19 (0.88)	
Female	3.93 (0.42)	4.22 (0.95)	
Total	3.87 (0.46)	4.20 (0.89)	− 2.40*

*p < 0.10 (one-tailed)

^a^Addictive behaviors scale ranged from 0 (never) to 6 (everyday)

^b^Addictive behaviors coded as categorical variable with 0 = never and 1 = at least once

^c^Behavioral intentions scale ranged from 1 (definitely will not) to 4 (definitely will)

^d^Psychosocial competencies scale ranged from 1 (strongly disagree) to 6 (strongly agree)

^e^Knowledge about addiction scale ranged from 0 (false) and 1 (true)

^f^Beliefs scale ranged from 1 (strongly disagree) to 6 (strongly agree)

The results of paired sample *t* tests on the parents participating in the program are shown in Table 3. Results revealed that parents showed a higher level of parenting skills and understanding of the relationship between addiction and parenting upon completion of the program (*t* (20) = − 2.03, *p* < 0.05). Results also showed that the parents at posttest reported a significantly higher level of positive identity and problem-solving capability (*t* (20) = − 2.29, *p* < 0.05). The findings provided evidence on the effectiveness of the training program for the parents.

**Table 3:** Mean scores (SD) of parent participants’ items tapping their main domains related to addiction at pretest and posttest periods, with results of paired sample *t* tests (*N* = 43)

Indicators	Pretest	Posttest	*t*
Parenting skills and knowledge on the relationship between addiction and parenting^a^			
Male	4.11 (0.75)	4.43 (0.86)	
Female	4.66 (0.67)	4.83 (0.68)	
Total	4.27 (0.80)	4.57 (0.77)	− 2.03*
Positive identity and problem-solving^a^			
Male	4.29 (0.98)	4.60 (0.63)	
Female	4.54 (0.56)	4.83 (0.80)	
Total	4.33 (0.95)	4.67 (0.70)	− 2.29*

*p < 0.05 (two-tailed)

^a^The scale ranged from 1 (strongly disagree) to 6 (strongly agree)

Subjective outcome evaluation findings are presented in Tables 4, 5, 6 and 7. Generally speaking, adolescents were highly satisfied with the curriculum content (Table 4) with positive responses ranging from 91.5 to 95.7%. Particularly, adolescents reported that the content design was very good (95.7%) and they liked the program very much (95.7%). Adolescents also expressed positive evaluations (ranging from 89.4 to 95.7%) toward the program implementers (see Table 5) who were very involved (97.9%) and cared for the participants (95.7%). Finally, adolescents reported that the program helped them in various domains such as facing family problems with positive attitude (69.6%), controlling (72.3%) and expressing (66.0%) their emotions, and developing a positive view of themselves (68.1%) (see Table 6).

**Table 4:** Summary of adolescents and parents’ positive responses (options 4–6) toward the curriculum

		Adolescents			Parents		
Items		*M* (SD)	*n*	%	*M* (SD)	*n*	%
1.	The objectives of the program are very clear	4.81 (1.15)	43	91.5	5.18 (0.59)	22	100
2.	The content design of the program is very good	4.79 (0.10)	45	95.7	5.27 (0.63)	22	100
3.	The activities were carefully arranged	4.81 (1.06)	43	91.5	5.23 (0.69)	22	100
4.	The classroom atmosphere was very pleasant	4.89 (0.98)	44	93.6	5.48 (0.68)	22	100
5.	There was much peer interaction among the students	4.89 (0.94)	44	93.6	5.24 (0.77)	22	100
6.	I participated in the class activities actively (including discussions, sharing and games, etc.)	4.85 (1.02)	43	91.5	5.18 (0.80)	22	100
7.	I was encouraged to do my best	4.87 (1.08)	44	93.6	5.14 (0.77)	22	100
8.	The learning experience enhanced my interests toward the program	4.81 (0.99)	45	95.7	5.27 (0.63)	22	100
9.	Overall speaking, I have a very positive evaluation of the program	4.89 (1.00)	43	91.5	5.46 (0.60)	22	100
10.	On the whole, I like this program very much	4.89 (0.94)	45	95.7	5.27 (0.63)	22	100

All items are on a six-point Likert scale with 1 = strongly disagree, 2 = disagree, 3 = slightly disagree, 4 = slightly agree, 5 = agree, 6 = strongly agree

**Table 5:** Summary of adolescents and parents’ positive responses (options 4–6) toward the program implementers

		Adolescents			Parents		
Items		*M* (SD)	*n*	%	*M* (SD)	*n*	%
1.	The implementer(s) had a good mastery of the program.	4.89 (1.01)	44	93.6	5.41 (0.59)	22	100
2.	The implementer(s) was (were) well prepared for the lessons.	4.89 (1.07)	42	89.4	5.50 (0.60)	22	100
3.	The teaching skills of the implementer(s) were good.	4.87 (0.92)	43	91.5	5.46 (0.60)	22	100
4.	The implementer(s) showed good professional attitudes.	4.85 (1.11)	42	91.3	5.50 (0.60)	22	100
5.	The implementer(s) was (were) very involved.	5.06 (0.89)	46	97.9	5.50 (0.67)	22	100
6.	The implementer(s) encouraged participants to participate in the activities.	5.00 (0.89)	42	91.3	5.50 (0.60)	22	100
7.	The implementer(s) cared for the participants.	5.06 (1.09)	45	95.7	5.46 (0.60)	22	100
8.	The implementer(s) was (were) ready to offer help to participants when needed.	5.06 (1.01)	44	93.6	5.41 (0.73)	22	100
9.	The implementer(s) had much interaction with the participants.	5.11 (1.03)	44	93.6	5.55 (0.74)	22	100
10.	Overall speaking, I have a very positive evaluation of the implementer(s).	4.98 (1.07)	42	89.4	5.68 (0.57)	22	100

All items are on a six-point Likert scale with 1 = strongly disagree, 2 = disagree, 3 = slightly disagree, 4 = slightly agree, 5 = agree, 6 = strongly agree

**Table 6:** Summary of adolescents’ positive responses (options 4–5) toward the program effectiveness

Items		*M* (SD)	*n*	%
1.	Enhanced my knowledge on the nature of addiction	4.04 (0.98)	33	70.2
2.	Eliminated my misconceptions about addiction	4.02 (0.87)	34	72.3
3.	Helped me to identify risk factors and protective factors of addictive behavior	4.00 (0.98)	33	70.2
4.	Helped me to understand the impact of various risk and protective factors on addiction	4.00 (0.98)	34	72.3
5.	Helped me to understand the influence of addictive behavior on my family members	4.06 (0.99)	32	68.1
6.	Enhanced my ability of emotional control	4.09 (0.86)	34	72.3
7.	Enhanced my ability to express emotions	4.02 (0.99)	31	66.0
8.	Helped me to face my family problems with a positive attitude	4.09 (0.94)	32	69.6
9.	Helped me to understand the influence of family factors on me	4.06 (1.01)	34	72.3
10.	Helped me to understand the influence of family factors on family relations	4.06 (1.07)	33	70.2
11.	Helped me to develop a positive view of myself	4.08 (0.95)	32	68.1
12.	Helped me to understand that although my parents have addictive behavior, I can still have good self-perception	4.00 (1.08)	34	72.3
13.	Helped me to discover my ability and potential	3.91 (1.10)	29	61.7
14.	Helped me to utilize internal resources to improve myself	4.00 (0.98)	31	66.0
15.	Helped me to make good use of human resources to improve myself	4.13 (0.94)	32	71.1
16.	Helped me to analyze problems and consider the feasible solutions	4.00 (0.94)	32	69.6
17.	Helped me to understand the importance of seeking assistance when necessary	4.09 (0.93)	35	74.5
18.	Helped me to understand the various ways to find assistance	4.09 (0.86)	34	72.3
19.	Helped me to overcome difficulties encountered when seeking assistance	3.96 (1.02)	31	66.0
20.	Promoted my overall development	4.00 (1.14)	33	70.2

All items are on a five-point Likert scale with 1 = not at all helpful, 2 = not very helpful, 3 = somewhat helpful, 4 = helpful, 5 = very helpful

**Table 7:** Summary of parents’ positive responses (options 4–5) toward the program effectiveness

Items		Mean (SD)	*N*	%
1.	Helped me to understand my children’s growth needs	4.14 (0.89)	15	68.2
2.	Helped me to understand the various ways to discipline my children	4.14 (0.77)	17	77.3
3.	Helped me to understand the influence of addictive behavior on family members	4.09 (0.87)	15	68.2
4.	Helped me to understand the relationship between disciplining children and addictive behavior	3.96 (0.79)	15	68.2
5.	Helped me to understand the importance of good relationships with children	4.19 (0.81)	16	76.2
6.	Helped me to understand good communication skills with children	3.95 (0.74)	15	71.4
7.	Helped me to learn how to deal with problems in a positive way	4.27 (0.77)	18	81.8
8.	Helped me to learn how to get along with my children	4.18 (0.80)	17	77.3
9.	Helped me develop my ability	3.72 (0.88)	12	54.5
10.	Helped me know how I can help my children grow	3.96 (0.65)	17	77.3
11.	Helped me to improve myself	4.09 (0.87)	17	77.3
12.	Helped me learn to encourage myself	4.13 (0.83)	18	81.8
13.	Encouraged me to be a responsible parent	4.18 (0.80)	17	77.3
14.	Encouraged me to be a capable parent	4.05 (0.79)	16	72.7

All items are on a five-point Likert scale with 1 = not at all helpful, 2 = not very helpful, 3 = somewhat helpful, 4 = helpful, 5 = very helpful

Parent participants were highly satisfied with the program content and implementers as evidenced by the positive responses across all items (100%) (see Tables 5 and 6). Parent participants also reported that after participating in the program, they better understood ways to discipline their children (77.3%) and deal with problems in a positive way (81.8%). They were also encouraged to be a more responsible (77.3%) and capable (72.7%) parent (see Table 7).

## Discussion

The present research evaluated the “Colorful Life - Multi-Addiction Intervention Program” targeted at the adolescents with addicted parents and parents with addiction in Hong Kong using objective and subjective outcome evaluation. Findings from the objective outcome evaluation with a pretest-posttest design demonstrated that after participating in all the sessions, adolescents’ beliefs in addiction changed in a positive direction (i.e., stronger beliefs that addiction is no good). Although there were no significant change in other domains (including addictive behavior, intention to engage in addictive behavior, or knowledge about addiction), a positive trend in the acquisition of psychosocial competencies was observed.

Several observations deserve our attention. First, the present program is one of the pioneer multi-addiction intervention programs for the adolescents with addicted parents and parent addicts. The unique feature of the adolescent program is that it was developed based on the syndrome model of addiction. Moreover, it adopts the positive youth development approach by aiming to strengthen adolescents’ intra- and interpersonal competencies among these high-risk youths. The aforementioned approach may possibly explain the positive trends observed in the acquisition of psychosocial competencies (e.g., being able to better express emotions, identifying personal strengths, and having a positive identity) among the adolescent participants. Our findings are in line with the previous studies that have shown the effectiveness of adopting the positive youth development approach in the prevention of problem behaviors, such as drug abuse among Hong Kong youths (Shek 2017). A large-scale curriculum-based positive youth development program Project P.A.T.H.S. (Positive Adolescent Training through Holistic Social Programmes) was developed and successfully implemented. Evidence demonstrated that the Program yielded long-term effects on preventing adolescent problem behaviors including the use of drugs and lowering intentions of participating in risk behaviors (Shek and Yu 2012; Shek 2017). Similarly, a multi-addiction intervention program targeted at primary school students has also demonstrated positive effects in enhancing students’ positive youth development constructs (Shek et al. 2016). The use of expressive arts to promote positive youth development within an addiction prevention context is a new breakthrough.

Second, the non-significant findings regarding addictive behaviors may be attributed to the floor effects (i.e., the level of addictive behaviors reported was quite low) which made the changes difficult to detect. Regarding knowledge about addiction, the majority of the traditional addiction prevention and intervention programs in Hong Kong have focused much on imparting knowledge about addiction. As students may easily access the Internet where they may acquire knowledge about addiction themselves, it is not surprising that the program did not help them acquire incremental knowledge on the matter, especially when our program adopted an expressive arts approach as opposed to the traditional uni-directional type of “lecturing” in most prevention and intervention programs.

For the parents, the findings from the objective outcome evaluation were generally positive. Participating in the program significantly helped parents to build a positive identity and improved their problem-solving skills. A non-significant positive trend was also found in the acquisition of parenting skills and psychosocial competencies after their participation in the program.

To complement the objective outcome evaluation findings, subjective outcome evaluation was also conducted. The findings from both adolescent and parent groups were generally positive. Particularly, the students liked the program and enjoyed the interactive activities. More importantly, the majority of the adolescents reported that the program helped them better understand the influence of addictive behavior on the family. They also reported enhancement in their ability to express and control their emotions. Also, the program helped them realize that even though their parents were addicts, they may still have positive self-perception. As for the parents, in terms of the program content and implementer attributes, almost all participants’ subjective evaluations were positive across all items. The results also revealed that the program was useful in introducing various ways to discipline their children and allowed them to understand the importance of maintaining a good relationship with their offspring. The program encouraged them to be a better parent and view themselves in a more positive light. Taken as a whole, the findings from the subjective outcome evaluation from both adolescent and parent participants are highly positive, and both parents and students perceived that the programs were beneficial to them.

According to the syndrome model of addiction (Shaffer et al. 2004) and positive youth development approach, while exposure and access to the object of addiction put one at risk, for instance, a parent that happens to be an addict, there are factors that help protect and reduce the likelihood of addiction (e.g., positive identity, social support, and emotional competence) (e.g., Shek et al. 2016; Yen et al. 2007). In line with previous findings that the nurturance of intrapersonal and interpersonal competencies may serve as protective factors against problem behaviors such as Internet addiction among youths (e.g., Shek and Yu 2016), the findings from the present study provided evidence for the effectiveness of the multi-addiction prevention and interventions based on the syndrome model as well as the value of adopting a positive youth development approach.

The present intervention is unique because few interventions in Hong Kong have articularly targeted at parent addicts. Specifically, our sessions aimed to highlight the relationship between parenting and addiction, to help parents better understand the needs of their offspring at different developmental stages, to equip them with parenting skills to strengthen parent-child relationship, and to strengthen parents’ own positive identity. Both quantitative and qualitative findings converged to demonstrate the effectiveness of the “Colorful Life” Intervention Program, particularly for the parent addicts in achieving the aforementioned objectives.

A review of literature also showed that many interventions for addicted parents are targeted at mothers, likely due to the assumption that the mothers are often the primary caregiver in the family (Silva et al. 2013). Research on the impacts of substance abusing fathers on family functioning and child development are grossly lacking (McMahon et al. 2008). Moreover, “fathering is an important, but largely neglected, treatment issue for drug-abusing men” (Söderström and Skårderud 2013, p. 32). This poses both knowledge and practice gap. Thus, we urge comprehensive approaches to guide research and interventions targeting both mothers and fathers suffering from addiction. Particularly, the interventions targeted at the parent addicts should include components that (a) foster empathic, contingent, and adjusted behaviors in parents; (b) help parenting conflicts resolution; (c) encourage development of reflective parental functioning; and (d) maximize the presence and effects of protective factors in the parent-child relationship in a broader context (Barrocas et al. 2016).

Our findings also provide evidence for the effective use of expressive arts in helping addicted parents and their children. The strengths of the use of expressive arts in creative counseling interventions have been well documented, such as fostering group cohesion, active participation, encouraging self-exploration and self-expression, and itself being a form of mind-body intervention. While the use of expressive arts in the treatment of substance abusers and relapse prevention in Hong Kong has gained popularity in recent years (Tam et al. 2016), it has rarely been used in multi-addiction prevention and interventions to help adolescents and their addicted parents. The findings from our study provided some insights into its application. While the interventions such as Tam et al.’s (2016) art-based relapse prevention group in Hong Kong were demonstrated to be effective, more research needs to be conducted to examine the effectiveness of expressive arts on high-risk adolescents who are not addicts themselves. Moreover, the authors suggested that expressive arts may serve as a useful approach for the individuals who are otherwise less verbal or sociable. As such, there may be individual differences in openness to and acceptance of the use of such approach in interventions that warrant further investigations.

To sum up, the evaluation findings from the present study provided evidence to support the effectiveness of the intervention and yielded practical implications. Nevertheless, readers should be cautious about the interpretation of the findings. First, the evaluation tools administered in this study were self-report in nature which may have impacted on the veracity of the findings. However, considering both practical and ethical concerns, this is the most feasible methodology. Future evaluation studies may consider adopting a more holistic evaluation approach by gathering perceptions from the family members of the participants as well. Second, the parents in the participant group were self-selected in terms of a strong motivation to improve their parent-child relations which brought them to the intervention. Thus, the present sample may represent a limited group of addicts who are willing to seek professional help and to invest in their family.

Our present work concludes with a call to develop, implement, and evaluate more multi-addiction interventions targeted at the offspring of addicts and parent addicts. Future research should be directed to the reduction of the effects of the risk factors, the promotion of the effects of protective factors at the individual, familial, and societal levels for the at-risk children and adolescents, and the strengthening of the role of both mothers and fathers in this care giving triad.
